# Viruses belonging to *Anelloviridae* or *Circoviridae* as a possible cause of chronic fatigue

**DOI:** 10.1186/s12967-020-02666-5

**Published:** 2020-12-14

**Authors:** Bjørn Grinde

**Affiliations:** Division of Mental and Physical Health, Norwegian Institute of Public Health, Skøyen, PO Box 222, 0213 Oslo, Norway

**Keywords:** ME/CFS, Viral infections, Anellovirus, Circovirus, Covid-19

## Abstract

Chronic fatigue often starts with an acute viral infection—as witnessed in the case of SARS-CoV-2—but indirect consequences of these infections are presumably the actual cause of the condition. As recently reviewed in this journal, the culprit could be a virus already present in the patient. The review covers several types of viruses, but concludes that the question is still open. The focus is on well known, pathogenic viruses for which there are ample diagnostic tools. I argue that there is one lesser-known group of viruses, the related anello- and circoviruses, which ought to be investigated. More or less everyone harbours at least one strain of these viruses in the blood, while not in the spinal fluid. They normally replicate at a low level, but their activity increases in an immune suppressed host; and there are cases where they do reach the brain. The initial infection could facilitate their access to the brain.

## Possible causes of chronic fatigue

There are several lines of investigation as to the aetiology of chronic fatigue syndrome (CFS), also known as myalgic encephalomyelitis. The arguably two most common are: one, immune system abnormalities such as chronic immune activation or autoimmunity; and two, as recently reviewed in the *Journal of Translational Medicine*, chronic viruses [1]. In both cases, several correlates are found, but none appears to give a satisfactory explanation for the condition. There are two problems with these correlates. For one, the parameters investigated tend to be limited to those with easily available diagnostic tools; and two, a correlate may be due to indirect effects of the actual causative factor. For example, if the aetiology is infectious, there would be an expected impact on in immunological parameters. On the other hand, if the immune surveillance is somehow compromised, there would be an expected increase in the titre (and detection rate) of chronic viruses.

What is generally accepted is that the condition in many cases (perhaps 50%) starts with an infection [2]. This is typically in the form of a viral-like flu with presumed viremia. The infection, however, is considered a triggering factor rather than the actual cause. A condition similar to CFS was noted as sequela in connection with the SARS epidemic, and again in connection with Covid-19 [3]. The observation accentuates the importance of finding the true culprit, but also offers an opportunity for research.

It makes sense to focus on reactivation of chronic, viral infections, as the typical initial agents are not expected to be around for the duration of the disease. Moreover, if other types of microbes were responsible, such as bacteria, it would presumably be easier to find the cause. The recent review covers most of the known, pathogenic viruses that have a strategy of lingering in the human host, including examples belonging to herpesviruses, enteroviruses, parvoviruses, retroviruses, and togaviruses [1]. I believe an additional diverse group of viruses ought to be investigated.

## Anello- and circoviruses

The family *Anelloviridae* is divided into 14 genera, including *Alphatorquevirus*, with the most studied, and ubiquitous, human Torque teno virus. The family *Circoviridae* comprises two genera, *Circovirus* and *Cyclovirus*. All these viruses have related, short (1.7-4.0 kb), circular, single-stranded DNA genomes. They form small, non-enveloped viral particles and are widespread in mammals and birds. Most humans apparently harbour several strains [4].

These viruses normally replicate at a low, but steady state. However, if the immune system is suppressed, their titres can rise to high levels in the blood [5]. Some strains are assumed to cause disease in animals, but until recently, they were considered completely benign in humans. Moreover, they were assumed to replicate solely outside the brain. Recent reports offer evidence for a rare presence in the brain, and a possible association with symptoms, in the case of both anelloviruses [6] and cycloviruses [7]. A neurological tropism of these viruses may be more common than expected as evidenced from studies of animals. Apparently, they can cause a condition resembling ME/CFS in pigs [8]. Certain strains of anelloviruses have been associated with multiple sclerosis, possibly in terms of harbouring peptide motives that could contribute to autoimmunity [9].

## Evidence for a role in CFS

The observation that perhaps half the cases of CFS starts with a severe infection, suggests that this infection facilitates an event that can also take place in its absence. Viral infections can suppress the immune system, for example, by reducing the production of interferons. This could lead to an increased titre of other viruses present in the patient. Viral infections can also compromise the blood brain barrier (BBB). Combined, these effects are likely to facilitate an access to the brain of viruses already present in the blood. Both effects are, for example, expected in the case of SARS coronaviruses. In severe infections with SARS-CoV-2, the virus is indeed found in the brain, but typically close to blood vessels, suggesting that the disease has facilitated a breach in the BBB [10]. The BBB normally protects the brain from viral infections, but once that barrier is passed, the brain appears to be particularly prone to virus-induced damage [1].

CFS displays a curious mixture of sporadic cases and occasional outbreaks [2]. The observation is congruent with the idea that particular strains of anello- or circoviruses are responsible. These viruses are highly infective, thus novel strains can spread easily. Either severe infections, which like covid-19 are epidemic in nature, other factors such as mental stress, or simply random events could cause the strains to infect the brain.

There appears to be a heritability factor in the case of CFS in that the condition runs in families [1]. A genetic predisposition is expected for most diseases. However, if the disease is caused by anello- or circoviruses, there is an additional explanation: These viruses can be transmitted from mother to child, and particular strains tend to run in families [4]. The mother to child transmission could also explain why mothers of adolescents with CFS more often display related symptoms than do the fathers. Certain strains would likely be more prone to cause CFS than others.

If CFS had been a normal consequence of an infection with a rare virus, one would expect a different epidemiological pattern. That is, the cases should cluster along possible infectious pathways. Most cases do not. The epidemiology fits better with the notion that the event leading to the disease is an unexpected crossing of the BBB by a virus present in many people.

Viruses affecting the brain are reputedly difficult to diagnose. Even in cases of encephalitis or meningitis, where there is reasonable evidence to suggest a viral aetiology, positive findings are obtained only in a fraction of cases. The problem may be partly due to the clinician not testing for the relevant virus, but the situation probably also reflects that viruses may cause clinical symptoms in the brain even if they replicate at a low level, and thus are difficult to detect.

Many patients with post-viral chronic fatigue recover within a few months. Those diagnosed with CFS are less likely to recover, perhaps because the diagnosis requires that the condition last for 6 months or more. If the immunological response is unable to clear the virus from the brain within 6 months, the virus may become chronically present there—as it tends to be outside the brain. In patients with CFS, the symptoms do fluctuate, and in many cases, the tendency is toward lasting improvements. These observations are in line with the idea that the brain is invaded by a virus tuned to chronic replication. The fluctuation of symptoms could reflect variations in viral activity, and, in some cases, the capacity of the patient to subdue the virus.

## Conclusions and future research

As previously suggested, CFS might be due to a normally non-pathogenic virus gaining access to the brain [11]. The cause of the event may be a severe infection by a more pathogenic agent. If so, anello- and circoviruses—or perhaps particular strains of these viruses—are likely candidates. These viruses are apparently well adapted to live in harmony with their hosts. The benign relationship might be breached either because the virus by accident enters unfamiliar territory, in the form of nervous tissue; or because the relevant strains have a recent zoonotic history.

The obvious next step is to look for these viruses in samples taken from the brain such as spinal fluid. PCR-based strategies would be the most sensitive, while metagenomic strategies cover a wider range of viruses. It should be noted that the project likely requires a sensitive technique, as the viruses presumably keep a low profile. Moreover, they may only be active in certain parts of the brain. One should preferably analyse samples taken soon after the initiation of the fatigue; as it is conceivable that the virus responsible causes damage, but is subsequently cleared from the brain.

Spinal fluid can be difficult to obtain, but it is also relevant to type the strains of anello- and circoviruses present in the blood. If the condition is caused by particular strains, one would expect a correlate with the strains present in the blood of patients compared to controls.

Generally, even a distinct presence of virus in samples taken from affected tissue is not conclusive as to the role of that virus in aetiology. For one, any severe disease may suppress the immune defence leading to an activation (and concomitant increased detectability) of chronic viruses; and two, many viruses use leukocytes for replication and these tend to aggregate in inflamed tissue. However, if certain viral strains correlate strongly with chronic fatigue, this suggests a role.

Ideally, one should have therapeutic agents that inhibit viral replication. If the agents also alleviate symptoms, there would be both a rational explanation for the fatigue and a treatment option. Due to their supposedly benign nature, the anello- and circoviruses have not been the focus of antiviral research. However, a recent report claims that a three-month treatment with the malaria medication artesunate did eliminate anelloviral DNA from blood cells in 62% of the cases [12]. If these viruses can be linked to chronic fatigue, more therapeutics are likely to appear.

## Data Availability

Not applicable.

## References

[1] Rasa S, Nora-Krukle Z, Henning N, Eliassen E, Shikova E, Harrer T (2018). Chronic viral infections in myalgic encephalomyelitis/chronic fatigue syndrome (ME/CFS). J Transl Med..

[2] Underhill RA (2015). Myalgic encephalomyelitis, chronic fatigue syndrome: an infectious disease. Med Hypotheses.

[3] Islam MF, Cotler J, Jason LA. Post-viral fatigue and COVID-19: lessons from past epidemics. Fatigue. 2020;8:61–9.

[4] Maggi F, Bendinelli M (2010). Human anelloviruses and the central nervous system. Rev Med Virol.

[5] Moen EM, Sagedal S, Bjoro K, Degre M, Opstad PK, Grinde B (2003). Effect of immune modulation on TT virus (TTV) and TTV-like-mini-virus (TLMV) viremia. J Med Virol.

[6] Eibach D, Hogan B, Sarpong N, Winter D, Struck NS, Adu-Sarkodie Y (2019). Viral metagenomics revealed novel betatorquevirus species in pediatric inpatients with encephalitis/meningoencephalitis from Ghana. Sci Rep..

[7] Tan le V, van Doorn HR, Nghia HD, Chau TT, Tu le TP, de Vries M, et al. Identification of a new cyclovirus in cerebrospinal fluid of patients with acute central nervous system infections. mBio. 2013;4:e00231–13. 10.1128/mBio.00231-13 PMC3684831 23781068

[8] Seeliger FA, Brugmann ML, Kruger L, Greiser-Wilke I, Verspohl J, Segales J (2007). Porcine circovirus type 2-associated cerebellar vasculitis in postweaning multisystemic wasting syndrome (PMWS)-affected pigs. Vet Pathol.

[9] Sospedra M, Zhao Y, zur Hausen H, Muraro PA, Hamashin C, de Villiers EM, et al. Recognition of conserved amino acid motifs of common viruses and its role in autoimmunity. PLoS Pathog. 2005;1:e41. 10.1371/journal.ppat.0010041 PMC1315278 16362076

[10] Marshall M (2020). How covid-19 can damage the brain. Nature.

[11] Grinde B (2008). Is chronic fatigue syndrome caused by a rare brain infection of a common, normally benign virus?. Med Hypotheses.

[12] Maltsev D (2020). The results of the study of important clinical aspects of TTV infection: spectrum of manifestations, association with minor immunodeficiencies, eficacy of artesunate. Immun Allergy Sci Pract.

